# Molecules for the millennium: how will they look? New drug discovery year 2000

**DOI:** 10.1054/bjoc.2000.1473

**Published:** 2000-11-22

**Authors:** E A Sausville, J I Johnson

**Affiliations:** Developmental Therapeutics Program, Division of Cancer Treatment and Diagnosis, National Cancer Institute, 6130 Executive Blvd. Suite 8000, Rockville, MD, 20852, USA

**Keywords:** drug discovery paradigm, molecular targets, anticancer therapeutics, in vitro screens

## Abstract

A new approach to cancer drug discovery targets molecules important in cancer pathogenesis. This approach is thought to be of greater promise than the antiproliferative screens which discovered cytotoxic agents and dominated cancer drug discovery for 60 years. However, one cannot lose sight of the fact that these targets exist in the cellular environment consisting of many additional influences on target function, and that effective drug treatment will take into account drug uptake, metabolism and elimination at the level of the cell as well as the organism. A key goal is to define for the new millennium a path to cancer drug discovery and development which accounts for the cancer cell phenotype in its totality rather than as arising solely from single molecular targets. The US National Cancer Institute maintains a cell-based drug discovery screen which can define a context for drug action in the milieu of more than 300 molecular targets and thousands of gene expression patterns which have been measured in the 60 human tumour cell lines which comprise the screening panel. The challenge of the millennium will be addressed by molecules active against defined targets but with selectivity of action occurring in the milieu of deregulated cancer cell biology in all its aspects. © 2000 Cancer Research Campaign http://www.bjcancer.com


*British Journal of Cancer* (2000) **83**(11), 1401–1404
© 2000 Cancer Research Campaign
doi: 10.1054/ bjoc.2000.1473, available online at http://www.idealibrary.com on IDEAL®

Millennium mini-review

### Molecules for the millennium: how will they look? New drug discovery year 2000

**EA Sausville and JI Johnson**

Developmental Therapeutics Program, Division of Cancer Treatment and Diagnosis, National Cancer Institute, 6130 Executive Blvd., Suite 8000, Rockville, MD 20852, USA

**Summary** A new approach to cancer drug discovery targets molecules important in cancer pathogenesis. This approach is thought to be of greater promise than the antiproliferative screens which discovered cytotoxic agents and dominated cancer drug discovery for 60 years. However, one cannot lose sight of the fact that these targets exist in the cellular environment consisting of many additional influences on target function, and that effective drug treatment will take into account drug uptake, metabolism and elimination at the level of the cell as well as the organism. A key goal is to define for the new millennium a path to cancer drug discovery and development which accounts for the cancer cell phenotype in its totality rather than as arising solely from single molecular targets. The US National Cancer Institute maintains a cell-based drug discovery screen which can define a context for drug action in the milieu of more than 300 molecular targets and thousands of gene expression patterns which have been measured in the 60 human tumour cell lines which comprise the screening panel. The challenge of the millennium will be addressed by molecules active against defined targets but with selectivity of action occurring in the milieu of deregulated cancer cell biology in all its aspects. © 2000 Cancer Research Campaign http://www.bjcancer.com

**Keywords:** drug discovery paradigm; molecular targets; anticancer therapeutics; in vitro screens

The pharmaceutical industry has invested in cancer drug discovery and development in recent years to an extent that vastly exceeds the level of corporate involvement even as late as 1990. According to the Pharmaceutical Research and Manufacturers of America (PhRMA), the aggregate corporate investment in cancer drug discovery can be estimated at $5 billion for the year 2000, and more than 350 agents are under corporate sponsorship as anti-cancer agents. This investment is predicated on the notion that advances in biology elucidating the molecular derangements in cancer cells will provide the basis for novel, safe and effective agents for cancer treatment. The underlying paradigm and areas of current focus have been reviewed comprehensively in a recent monograph by Garrett and Workman (Garrett and Workman, 1999). We shall consider here counterpoints to and ramifications of this paradigm to expand the discussion of what qualities molecules for a new millennium of cancer treatment might possess. We shall illustrate as case studies the exceptional instances where current molecules under study approach this ideal, or illustrate potential bumps in the road.

A key tenant of the assumed wisdom leading us down a new path for cancer drug discovery and development is that molecules with pathogenic significance in the *causation* of cancer will be key targets in the useful *treatment* of cancer. The anti-*bcr-abl* directed protein kinase antagonist STI-571, a drug molecule crafted to be a specific kinase inhibitor, has emerged as a 'poster child' for this new approach (Drucker and Lydon, 2000). STI-571 is a prototype that hopefully can be generalized to other drug molecules in addressing haematologic and paediatric neoplasms which utilize translocation-related fusion proteins as a basis for their existence. Yet, it is very unusual in adult oncology that a single target can be tied unequivocally to a cancer's pathogenesis. It is further unusual in adult oncology for a cancer's susceptibility to the therapeutic agent to be uniquely biased, as normal human cells do not possess anything quite like *bcr-abl*. While it is true that c-*abl* is a ubiquitously expressed portion of *bcr-abl*, it appears to have a very distinct set of cellular functions and a limited set of substrates in comparison to *bcr-abl*. Common adult solid tumours actually contain dozens of interacting targets which deregulate cell cycle progression, convey cellular immortality (for example, by activating telomerase), have disabled cell death-promoting mechanisms, and in the majority of lethal tumours have become functionally enabled to induce new blood vessels, invade and metastasize. Thus, a key feature of a hoped for 'molecule of the millennium' will be the ability to address multiple deranged targets in a way that is qualitatively different than current cytotoxics, whose value arises from the *cellular response* to the drug rather than from the nature of the drugs' targets.

How might these properties be 'built-in' to drugs of the millennium? One approach, outlined by Kaelin (Kaelin, 1999), is to define molecules which are active only in the context of the genetically altered environment of the tumour cell. That is, one must design agents whose actions address perhaps not a single target, but rather, a molecular context for a target 'process'. The relevant target process for cancer treatment may depend on a number of molecules, and the challenge will be in discerning the best way of perturbing the process. For example, cancer cells possess damaged cell cycle checkpoints as a key feature in tolerating and indeed utilizing genetic plasticity to create ever more successful neoplastic cells. It is therefore desirable to define therapeutic molecules that act in a manner analogous to 'synthetic lethal' mutations, which result in non-viable organisms only in the

*Received 7 August 2000*
*Accepted 8 August 2000*

*Correspondence to:* EA Sausville context of damaged checkpoint mutations. By this approach, drug discovery screens might be devised to detect molecules active only in cells bearing mutations in the various checkpoint-regulated pathways. Friend, Hartwell and colleagues have devised a screen which detects compounds (Simon et al, 2000) with differential activity in yeast engineered to possess such checkpoint alterations, and data emerging from this effort are available in part through http://dtp.nci.nih.gov. As with any screen, however, these are only 'leads' whose relevance to mammalian tumour cell biology must now be explored and exploited. Other types of 'synthetic lethal' screens might be conceived, e.g., molecules active only in the context of an activated 'mutation' phenotype, or active in the context of deregulated E2F (Kaelin, 1999).

An extension of this way of thinking is to recognize that a molecular target of one type, e.g., which promotes cell cycle deregulation, exists in a cancer cell in the context of other molecules responsible for a target-directed drug's uptake, intracellular metabolism and elimination. An example in the most elementary sense of this concept is afforded by 5-fluoropyrimidines. Studies by Danenberg and colleagues have illustrated the potential importance of the level of thymidylate synthase (TS) and DNA repair enzymes, such as ERCC1, in conveying the likelihood of clinical responses to 5-fluorouracil (5FU) and cis-platinum-based regimens (Metzger et al, 1998). More recently, it has likewise been elucidated that the expression or activity of dihydropyrimidine dehydrogenase (DPD) is a key determinant of susceptibility to fluoropyrimidines (Salonga et al, 2000). Therefore, the real 'target' of 5FU is not simply TS, but the milieu created by a particular cell type's susceptibility to thymineless death (perhaps governed by p53 and cell cycle checkpoint-related pathways), plus its TS and DPD levels, not to mention the nucleoside salvage pathways which allow formation of FdUMP in the first place. These studies emphasize that elucidation of novel agents to perturb target molecules must operate in a cellular milieu, where the substrate of the target's action and indeed the drug molecule, are variables that must be effectively addressed in correcting the consequences of targeted effects.

A recently defined example where drug metabolism must be considered in relation to the design of drugs to effect a growth regulatory molecule is the elucidation that the action of geldanamycins, which target hsp90-binding signal transducing molecules (Whitesell et al, 1994; Stebbins et al, 1997) can be influenced by expression of DT-diaphorase (Kelland et al, 1999). It is thus possible that the real 'target' of geldanamycin is the relationship between 'levels' or activity of hsp90-related signalling systems in relation to the consequences of diaphorase action on geldanamycins. Elucidation of the cellular disposition of bound drug, therefore, becomes of great importance in how to design agents related to target action or to optimize the cellular events effected by a drug.

These circumstances lead us to challenge the assertion that there is little value in understanding the action of novel cytotoxic compounds, irrespective of how exotic their point of origin may be (Garrett and Workman, 1999). For example, the novel cytotoxic agent, ET 743 (ecteinasceidin), causes a unique DNA lesion (Pommier et al, 1996). Early clinical studies have suggested some activity on the part of ET 743 in soft tissue and osteosarcomas, but at the cost of noteworthy toxicity (Taamma et al, 1998). The challenge to natural product and synthetic chemists is to team with biologists to understand how to steer the unique cytotoxic mechanism of ET 743 to where it is useful with an enhanced therapeutic index. In fact, obstinately persisting in clinical development schemes without considering the possibility of molecular refinement could doom an otherwise potentially useful agent.

Discovery screens for molecules of the millennium must, therefore, be designed with much deliberation as to the cellular contexts of targets residing in the selected tumour cells. In this regard, databases of target expression as a function of cancer cell type will be invaluable. The Cancer Genome Anatomy Project (CGAP) is creating publicly accessible databases of differentially expressed genes in various cell types (http://www.ncbi.nlm.nih.gov/CGAP/). Another publicly accessible database is the NCI in vitro anticancer drug screening database. The methods and operation of this screen have recently been altered to increase capture of cell information while improving cost-effectiveness. The drug screen currently utilizes an in vitro prescreen consisting of three human tumour cell lines (breast MCF7, CNS SF-268, and lung NCI-H460) against which potential chemotherapeutic agents are tested at a single dose for a 48-hour exposure period. Test agents which inhibit the growth of at least one of the cell lines to 32% or less of control cell growth are tested against a panel of 60 human tumour cell lines over a 5-dose range of one-log dilutions. This in vitro assay system has been described previously (Monks et al, 1991; Paull et al, 1995; Alley et al, 1998), and the most current information concerning this test system is catalogued on the Developmental Therapeutics Program, NCI Web site (http://dtp.nci.nih.gov). The NCI in vitro cell screening data can be used as a basis for assessing initial mechanisms of action through computational database searching. For example, COMPARE is a pattern-recognition algorithm which compares the effect of a test agent on the 60 cell line panel to the database of both standard agents of known anti-proliferative mechanism, as well as that of more than 77 000 compounds which have been evaluated in the screen. The use of this database to discover novel associations of drug chemotypes with molecular mechanisms has been previously described (Monks et al, 1997).

Initial studies with the MDR-1 drug resistance protein encouraged the notion that profiling of the cell line panel for a target could lead to the definition of structure activity through or in relation to the target (Wu et al, 1992). More than 300 such single molecular target determinations are currently either underway or have now been carried out, mostly through highly valuable collaborations with academia and industry, and are listed on http://dtp.nci.nih.gov. COMPARE is utilized to determine if the pattern of cellular sensitivity and resistance for a test compound can be related to a measured molecular target. The molecular target COMPARE has identified, for example, *ras* inhibitors (Koo et al, 1996), DT-diaphorase correlates (Fitzsimmons et al, 1996), and thioredoxin inhibitors (Kunkel et al, 1997). As additional collaborations are fostered and additional targets measured, patterns of activity from the entire database of results from the in vitro 60 cell line panel are compared to the target database in an effort to identify new leads for development. Data from the in vitro screen have also been analysed with the help of DISCOVERY (Weinstein et al, 1997), which uses a clustering algorithm to group compounds by cellular response patterns. The clustered correlation feature of DISCOVERY is used to produce a map of the relation between chemical compounds evaluated in the 60 cell line assay and molecular targets which have been measured in the cells.

 *© 2000 Cancer Research Campaign*

A key recent evolution of the screening data has been the availability of gene expression patterns for thousands of expressed genes through NCI's collaboration with Brown and Botstein (Ross et al, 2000), and Weinstein (Scherf et al, 2000). The public availability of these data through, for example, http://dtp.nci.nih.gov, offers a way of aligning the presence of various chemotypes to patterns of gene expression. Intrinsic ability of a given chemotype to cause an anti-proliferative effect may be aligned with efforts to design structures with the capacity to affect additional targets present in the desired cell type.

Thus, a hoped for 'molecule of the millennium' might act to affect an important target pathway, e.g., the *ras* pathway, not by focusing on a feature such as its dependence on the promiscuously expressed farnesyl transferase modification, but by focusing on the essence of '*ras*-ness', namely the ability of the drug to interact persistently with its proliferation-promoting targets, e.g., *raf* or P13-kinase. The drug molecule might have been detected by a functional screen which defined alteration of *raf*-related or P13-kinase signal output as a criterion for success. The drug will have been further modified to promote the successful uptake or distribution to, for example, visceral solid tumours (pancreas, lung, colon, etc.) where the biologic data is compelling for a role for *ras* in promoting malignant cell behaviour. The molecule will have been crafted to avoid or de-emphasize facile metabolic deactivation, or will have been selected to possess prolonged residence time at the target. Appreciation of the metabolic milieu in which the drug must function will have been gleaned by considering the frequency and type of genes in databases such as CGAP and related efforts.

This last point raises another issue in selecting molecules for further optimization as cancer therapeutics. A common dictum in the development of small molecules for non-cancer indications is that molecules with high affinity for their particular target but which act in a biochemically reversible mechanism should be the goal of drug discovery paradigms. Affinity constants of nM are to be desired. Molecules which cause covalent modification of target proteins, e.g., Michael acceptors or molecules with 'alkylating' potential should be avoided. Since cancer treatments must, as described above, operate in a cellular environment relevant to several molecular targets to convey a persistent cytostatic or very differentially cytotoxic effect, we must consider that some rethinking of these precepts may be in order to address the special case of cancer therapy-directed agents. Specifically, a very tight binding inhibitor that is functionally irreversible or indeed covalent may not be such a bad idea, provided that the binding occurs in an appropriately specific context. The value of this approach would then be to allow more intermittent dosing and thus a potentially augmented therapeutic index. Certain conventional agents, e.g., deoxycoformycin vs. adenosine deaminase, or investigational agents, e.g., $O^6$-benzyl guanine vs. alkyl-guanine alkyl transferase, demonstrate that selectivity of action is possible even with such molecular features. Certain protein kinase antagonists likewise are covalent tight binding inhibitors with marked selectivity for targets (Fry, 1999). In fact, a covalent or kinetically irreversible interaction with a specific target may be yet one more way of augmenting drug selectivity to the range of targets expressed in the cancer cell milieu.

The development of irreversible or ultra-tight binding inhibitors also recognizes that an ultimate goal of cancer treatment for the major fraction of afflicted patients is reduction of cell mass. As the greatest fraction of cells of the average solid tumour may not be in an actively proliferating phase of the cell cycle at the time of treatment, such tight binding inhibitors may occupy their binding sites and therefore be available to afford a useful therapeutic effect at the time the cell next attempts cycle re-entry. By this view, an additional challenge for the millennium will be discerning targets whose efficient ligation to a small molecule will lead to a signal for apoptosis, as a result of their structure or altered function in the liganded state. While it could be argued that this paradigm is suspiciously similar to that which underlies the action of DNA-directed alkylating agents, we must remember that these drugs actually do work, albeit in the minority of diseases. One would profitably consider the characteristics which afforded these drugs their success, and attempt to emulate their properties, but with a range of novel targets differentially expressed in tumour cells.

A final topic area is the important role of the cancer cell micro-environment in defining a basis for potential differential susceptibility to new agents. Other authors have stressed aberrant micro-circulation and dependence on neovascularization ubiquitous in successful tumours (Hahnfeldt et al, 1999; Ramanujan et al, 2000). It is still not clear how the expression of biochemical targets in the matrix, consisting of altered, proteolysed basement membrane, clotting cascade elements and remnants, and shed cell surface antigens and receptors can be systematically defined in primary and metastatic lesions from various tumour types. This micro-environment could serve as a barrier to drug penetration, or could actually be the basis for devising strategies for novel tumour targeting properties again 'built into' the drug's design. The 'molecule of the millennium' would be designed with the tumour micro-environment in mind and potentially use its characteristics to advantage. This strategy would further promote a marriage between traditional small molecule drug discovery paradigms and biologically based selection strategies such as the use of phage display to define binding motifs with relevance to tumour matrix components.

It is clear that we are poised upon the threshold of a new age of cancer therapeutics predicated on exploiting the *differences* between normal and cancer cells. We will depend for accurate depiction of these therapeutic opportunities on the molecules that define successful tumours as they occur in the human host, and not merely the ubiquitously deranged common lesions that *cause* the cancer, as revealed by experiments in model systems. Design of successful drugs must take into account aspects of a tumour cell's molecular contexts, metabolism and architecture. Development schemes to emphasize 'real time' confirmation in pre-clinical models as well as in early clinical trials that a drug is having the desired effect on its intended target and delivery to target tumour cell populations would be optimal and should be seriously considered as adding value and not 'frosting' on development plans. For molecules with an intended cytostatic effect, such information could be key in deciding to move a molecule to initial or more advanced (post Phase I) clinical testing. In this way there will be a seamless linking of the opportunities dreamed of at a drug molecule's initial conception with optimal development and use.

### REFERENCES

Alley MC, Scudiero DA, Monks A, Hursey ML, Czerwinski MJ, Fine DL, Abbott BJ, Mayo JG, Shoemaker RH and Boyd MR (1998) Feasibility of drug

© 2000 Cancer Research Campaign screening with panels of human tumor cell lines using a microculture tetrazolium assay. *Cancer Res* **48**: 589–601

Drucker BJ and Lydon NB (2000) Lessons learned from the development of an abl tyrosine kinase inhibitor for chronic myelogenous leukemia. *J Clin Invest* **105**: 3–7

Fitzsimmons SA, Workman P, Grever M, Paull KD, Camalier R and Lewis AD (1996) Reductase enzyme expression across the National Cancer Institute tumor cell line panel: correlation with sensitivity to mitomycin C and E09. *J Natl Cancer Inst* **88**: 259–269

Fry DW (1999) Inhibition of the epidermal growth factor receptor family of tyrosine kinases as an approach to cancer chemotherapy: progression from reversible to irreversible inhibitors. *Pharmacol Ther* **82**: 207–218

Garrett MD and Workmann P (1999) Discovering novel chemotherapeutic drugs for the third millennium. *Eur J Cancer* **35**: 2010–2030

Hahnfeldt P, Panigrahy D, Folkman J and Hlatky L (1999) Tumor development under angiogenic signaling: a dynamical theory of tumor growth, treatment response, and postvascular dormancy. *Cancer Res* **59**: 4770–4775

Kaelin WG (1999) Choosing anticancer drug targets in the post genomic era. *J Clin Invest* **104**: 1503–1536

Kelland LR, Sharp SY, Rogers PM, Myers TG and Workman P (1999 DT-Diaphorase expression and tumor cell sensitivity to 17-allylamino, 17-demethoxygeldanamycin, an inhibitor of heat shock protein 90. *J Natl Cancer Inst* **91**: 1940–1949

Koo H, Monks A, Mikheev A, Rubinstein L, Gray-Goodrich M, McWilliams MJ, Alvord WG, O HO, Gazdar AF, Paull KD, Zarbl H and Vande Woude GF (1996) Enhanced sensitivity to 1-β-D-arabinofuranosylcytosine and topoisomerase II inhibitors in tumor cell lines harboring activated *ras* oncogenes. *Cancer Res* **56**: 5211–5216

Kunkel MW, Kirkpatrick DL, Johnson JI and Powis G (1997) Cell line-directed screening assay for inhibitors of thioredoxin reductase signaling as potential anti-cancer drugs. *Anticancer Drug Des* **12**: 659–670

Metzger R, Leichman CG, Danenberg KD, Danenberg PV, Lenz HJ, Hayashi K, Groshen S, Salonga D, Cohen H, Laine L, Crookes P, Silberman H, Baranda J, Konda B and Leichman L (1998) ERCC1 mRNA levels complement thymidylate synthase mRNA levels in predicting response and survival for gastric cancer patients receiving combination cisplatin and fluorouracil chemotherapy. *J Clin Oncol* **16**: 309–316

Monks A, Scudiero D, Skehan P, Shoemaker R, Paull K, Vistica D, Hose C, Langley J, Cronise P, Vaigro-Wolff A, Gray-Goodrich M, Campbell H, Mayo J and Boyd M (1991) Feasibility of a high-flux anticancer drug screen using a diverse panel of cultured human tumor cell lines. *J Natl Cancer Inst* **83**: 757–766

Monks A, Scudiero DA, Johnson GS, Paull KD and Sausville EA (1997) The NCI anti-cancer drug screen: a smart screen to identify effectors of novel targets. *Anticancer Drug Des* **12**: 533–541

Paull KD, Hamel E and Malspeis L (1995) Prediction of biochemical mechanism of action from the *in vitro* antitumor screen of the National Cancer Institute. In: Foye WO (ed), *Cancer Chemotherapeutic Agents*. American Chemical Society, Washington, DC, pp. 9–45

Pommier Y, Kohlhagen G, Bailly C, Waring M, Mazumder A and Kohn KW (1996) DNA-sequence and structure-selective alkylation of guanine N-2 in the DNA minor groove by ecteinascidin 743, a potent antitumor compound from the Carribean tunicate *Ecteinascidia turbinata*. *Biochemistry* **35**: 13303–13309

Ramanujan S, Koenig GC, Padera TP, Stoll BR and Jain RK (2000) Local imbalance of proangiogenic and antiangiogenic factors: a potential mechanism of focal necrosis and dormancy in tumors. *Cancer Res* **60**: 1442–1448

Ross DT, Scherf U, Eisen MB, Perou CM, Rees C, Spellman P, Iyer V, Jeffrey SS, Van de Rijn M, Waltham M, Pergamenschikov A, Lee JC, Lashkari D, Shalon D, Myers TG, Weinstein JN, Botstein D and Brown PO (2000) Systematic variation in gene expression patterns in human cancer cell lines. *Nat Genet* **24**: 227–235

Salonga D, Danenberg KD, Johnson M, Metzger R, Groshen S, Tsai PI, Levy HJ, Leichman CG, Leichman L, Niasio RB and Danenberg PV (2000) Colorectal tumors responding to 5-fluorouracil have low gene expression levels of dihydropyrimidine dehydrogenase, thymidylate synthase, and thymidine phosphorylase. *Clin Cancer Res* **6**: 1322–1327

Scherf U, Ross DT, Waltham M, Smith LH, Lee JK, Tanabe L, Kohn KW, Reinhold WC, Myers TG, Andrews DT, Scudiero DA, Eisen MB, Sausville EA, Pommier Y, Botstein D, Brown PO and Weinstein JN (2000) A gene expression database for the molecular pharmacology of cancer. *Nat Genet* **24**: 236–244

Simon JA, Szankasi P, Nyugen DK, Ludlow C, Dunstan HM, Roberts CJ, Jensen EL, Hartwell LH and Friend SH (2000) Differential toxicities of anti-cancer agents amoung DNA repair and checkpoint mutants of *Saccharomyces cerevisiae*. *Cancer Res* **60**: 328–333

Stebbins CE, Russo AA, Schneider C, Rosen N, Hartl FU and Pavletich NP (1997) Crystal structure of an Hsp90-geldanamycin complex: targeting of a protein chaperone by an antitumor agent. *Cell* **89**: 239–250

Taamma A, Riofrio M, Cvitkovic E, Meely K, Mekranter B, Jimeno J, Cmaeron L, Beijnen JH and Misset JL (1998) Ecteinascidin-743 (ET-743) 24 hours continuous infusion (CI): Clinical and pharmacokinetic Phase I study in solid tumor patients (Pts). Preliminary results. *ASCO Proc* #890.

Weinstein JN, Myers TG, O'Connor PM, Friend SH, Fornace AJ Jr, Kohn KW, Fojo T, Bates SE, Rubinstein LV, Anderson NL, Buolamwini JK, van Osdol WW, Monks AP, Scudiero DA, Sausville EA, Zaharevitz DW, Bunow B, Viswanadhan VN, Johnson GS, Wittes RE and Paull KD (1997) An information-intensive approach to the molecular pharmacology of cancer. *Science* **275**: 343–349

Whitesell L, Mimnaugh EG, De Cosla B, Myers CE and Neckers LM (1994) Inhibition of heat shock protein HSP90-pp60V-src heteroprotein complex formation by benzoquinone ansamycin: roles for stress proteins in oncogenic transfection. *Proc Natl Acad Sci USA* **91**: 8324–8328

Wu L, Smythe AM, Stinson SF, Mullendore LA, Monks A, Scudiero DA, Paull KD, Koutsoukos AD, Rubinstein LV and Boyd MR (1992) Multidrug-resistant phenotype of disease-oriented panels of human tumor cell lines used for anticancer drug screening. *Cancer Res* **52**: 3029–3034

 *© 2000 Cancer Research Campaign*

